# Integrated Palliative Outcome Scale for People with Dementia: easy language adaption and translation

**DOI:** 10.1186/s41687-022-00420-7

**Published:** 2022-02-15

**Authors:** Frank Spichiger, Anita Keller Senn, Thomas Volken, Philip Larkin, Andrea Koppitz

**Affiliations:** 1 School of Health Sciences Fribourg, HES-SO | University of Applied Science and Arts Western Switzerland, Fribourg, Switzerland; 2 Institute of Nursing, Faculty of Biology and Medicine, UNIL | University of Lausanne, Lausanne, Switzerland; 3 Department of Endocrinology and Diabetology, KSW | Cantonal Hospital Winterthur, Winterthur, Switzerland; 4 Institute of Health Science, School of Health Professions, ZHAW, Zurich University of Applied Science, Winterthur, Switzerland; 5 CHUV, Lausanne University Hospital, Lausanne, Switzerland

**Keywords:** PROMS, Translation, Validation studies, Dementia

## Abstract

**Background:**

In this article, we report the cultural adaption and translation of the Integrated Palliative Care Outcome Scale for People with Dementia (IPOS-Dem) into a Swiss-German easy language version for proxy assessment of people with dementia living in Swiss nursing homes. The Swiss-German easy language version of the IPOS-Dem was developed and culturally adapted in a six-phase process from the German IPOS-Dem using recommended guidelines. With nursing home staff and laypeople, the conceptual definition and relevance of IPOS-Dem items were established during phase I. Phase II encompassed the completion of forward translations. Independent native speakers blind to the original scale translated and back-translated the Swiss-German easy language version. The resulting IPOS-Dem version was then blindly back-translated in phase III. Experts reviewed all resulting translations in phase IV to produce a pre-final IPOS-Dem version. Finally, the phase V cognitive debriefing involved two focus groups assessing the pre-final IPOS-Dem version. Phase V included cognitive interviews with laypeople (*n* = 2), family members of those with dementia (*n* = 4) and staff from different care contexts (*n* = 12).

**Results:**

Using easy language specialists yielded a clinically relevant, comprehensive and understandable translation. In addition, face and content validity for the easy language version were established in the cognitive interviews.

**Conclusions:**

With an easy language IPOS-Dem, all frontline staff and family members can be empowered to communicate their observations after caring interactions. Enhanced clinical communication with easy language tools shows the potential for research and clinical applications. In addition, attentive use in scales of easy language communication may foster increased engagement with untrained laypeople in clinical and care research.

**Supplementary Information:**

The online version contains supplementary material available at 10.1186/s41687-022-00420-7.

## Introduction

People living with dementia in nursing homes and the community are at high risk of serious health-related suffering [1]. Since there are no curative treatment options for dementia, focussing on a palliative care approach is indicated [2]. Palliative care is a holistic approach to care. The needs and concerns of a person with any disease that does not respond to disease-modifying treatment are managed with priority [3]. Therefore, assessing needs and concerns is a prerequisite for providing high-quality care for people with dementia [3]. However, the timely and structured assessment of needs and concerns in Swiss nursing homes is rare [4, 5]. Furthermore, the current assessment processes and instruments are biased towards behavioural, psychiatric symptoms instead of needs and concerns. Further, the regulations attached to the assessment do not formally permit most frontline workers to document their observations [6, 7].

The Integrated Palliative Care Outcome Scale for People with Dementia (IPOS-Dem) is a brief and multi-dimensional instrument for proxy needs and concerns screening by nursing staff [8]. It provides caregivers with an overview of the outcomes achieved and may determine whether a treatment is worthwhile, indicating which services and interventions are the most adequate. Care staff score 27 items in the IPOS-Dem with a Likert scale (0 to 4) [9]. Hodiamont et al. [10] translated the IPOS-Dem from English to German for German health professionals and family members.

Swiss dialects cannot be easily understood by German-speaking people [11]. People living in the Swiss-German region use a variety of Alemannic dialects in all day-to-day interactions [12]. The Alemannic dialects of Switzerland have a high presence; they are only exceptionally substituted with standard German. Their prevalence in spoken communication and public media sets them apart from Alemannic dialects spoken in Austria, Germany, and northern Italy. Officially, Switzerland is a four-language state, where French, German, Italian, and Romansh are used for written and spoken communication [11]. Swiss standard German, however, is usually spoken only on request and understood by the majority of people living in the Swiss-German region. It is much more accessible to other people speaking German, but subtle differences to standard German remain [12].

Up to 70% of frontline staff working in Swiss nursing homes have a migratory background [13]. As a result, Swiss nursing homes commonly employ a heterogenous mix of staff to care for people with dementia—this includes untrained personnel, volunteers, and interns with varying degrees of literacy and language skills. Papadakus [14] found that the linguistic accessibility of instruments and scales for patient assessment is lacking in the development of such instruments. Worldwide, 15 to 20% of the population has reading difficulties [15].

A standardised ruleset for ‘Leichte Sprache’ (easy language or easy-read) has been agreed upon for German-language regions [16]. Plain language in care and medical settings has become popular in explanatory texts and self-management [17]. However, linguistic assessment and critique mainly focus on legal texts [18]. Easy language is a further development of plain language texts. Easy language enhances text accessibility and readability for people with and without reading difficulties compared to plain language. Easy language has recommendations for, e.g., word difficulty and precision, use of numbers in the text and sentence length [16]. International resources for easy language are available from https://www.inclusion-europe.eu/easy-to-read/ and for plain language at the European Publications Office [19]. Therefore, for our study involving nursing homes in the German-speaking part of Switzerland with heterogeneous frontline staff, we translated the original IPOS-Dem to easy language for the Swiss context.

## Aims

In this study, the German IPOS-Dem version was translated and culturally adapted into easy German for the Swiss nursing home context and frontline staff. The secondary aim was to describe the discrepancies and differences that occurred during adaption compared to previous research.

## Methods

**Fig. 1:**
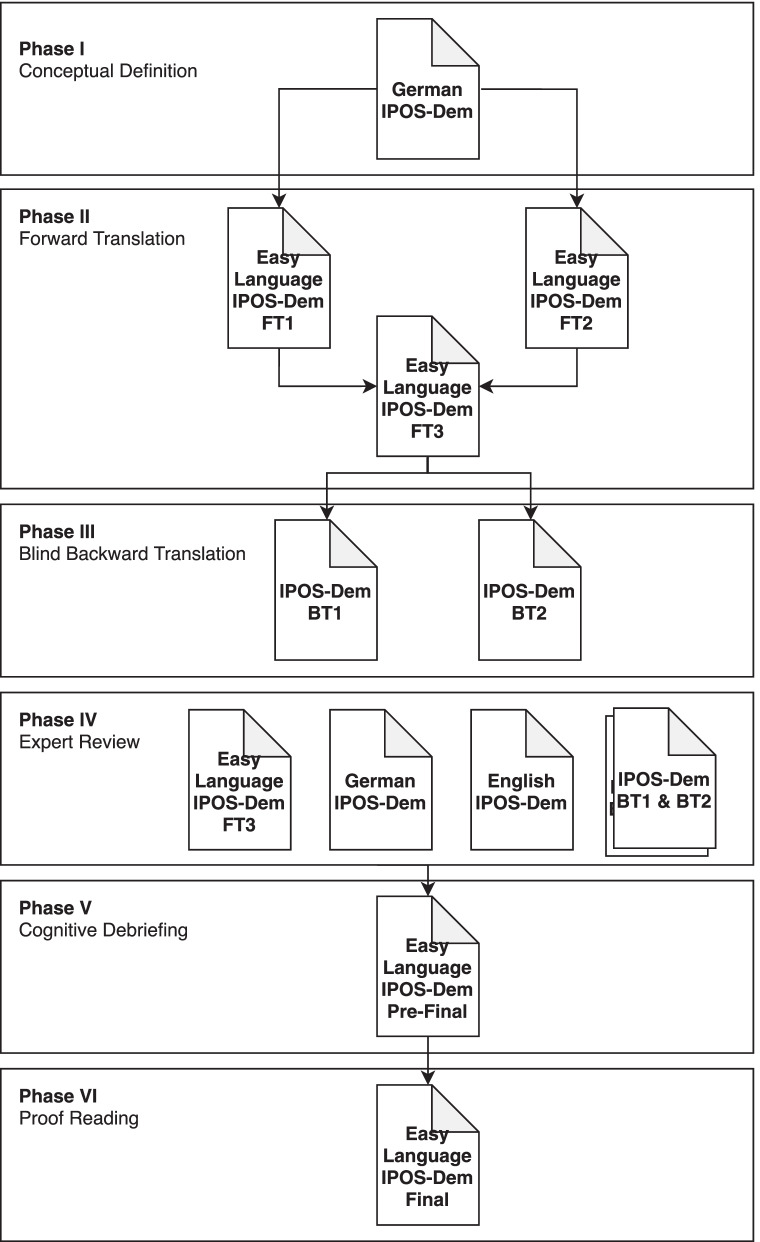
Phases and documents produced in the IPOS-Dem translation and adaptation to ‘Leichte Sprache’. Abbreviations: BT1 and BT2: backward translations; FT1, FT2 and FT3: forward translations 1, 2 and 3, respectively; IPOS-Dem: Integrated Palliative Care Outcome Scale for People with Dementia

Our linguistic and cultural adaption was guided by an internationally defined methodology [20] and a stepped process for translation [21, 22]. Our translation was undertaken between August 2020 and April 2021. Figure 1 illustrates the six-phase process for translation and adaption that we followed. The study was conducted in multiple centres in the eastern Swiss-German part of Switzerland. In the first translation, we involved certified translators for easy language from ProInfirmis. Scientific experts and laypeople consulted with us later in the adaption in offsite, online meetings due to public health restrictions. We applied multiple methods during this process: forward and backward translation, expert focus groups and cognitive interviews with staff from different settings in which they work with people with dementia. Interview data were analysed via thematic analysis. Psychometric testing (stage VII proposed by Antunes et al. [23]) will be detailed separately.

### Phase I: Conceptual definition

We collated an overview of the literature related to current clinical practices in health-related quality of life measurement for people with dementia in Swiss nursing homes. We examined processes and systematic assessment behaviours in frontline staff. Furthermore, we interviewed our clinical partners on the use of routine assessment instruments, outcome measurements and health-related quality of life measures they know or use. With six participants, we conducted formal and informal discussions on the relevance of the IPOS-Dem items concerning their key concepts in August 2020. The formal discussions were recorded, and the anonymised transcripts were thematically analysed.

### Phase II: Forward translation

Three translators were involved in the forward translation process. Two certified translators for easy language with no clinical background wrote the first forward translation (FT1) from the German IPOS-Dem. Translator three had a nursing background with a specialisation in family nursing. Translator three wrote forward translation two (FT2) from the German IPOS-Dem. Finally, a research team member synthesised and consolidated the forward translations (FT1 and FT2) into forward translation three (FT3).

### Phase III: Backward translation

The translators involved in this phase were blinded to the original English IPOS-Dem and intermediary German IPOS-Dem. Both translators are native-English-speaking American nurse educators living and working in Switzerland who are proficient in German. The translators were involved in the backward translation process of FT3. Each produced a German backward translation (BT1 and BT2). Five participants later reviewed and compared the German IPOS-Dem, BT1, BT2 and FT3.

### Phase IV: Expert review

An expert group (*n* = 9) held two online meetings in March 2021 to review and discuss the different versions: the original English IPOS-Dem, the German intermediary IPOS-Dem and FT3. Table 1 provides a short description of the expert group members. The meeting was facilitated by a research team member. Based on this review of the documents, the panel agreed upon and ratified the pre-final version.

**Table 1:** Demographics of study participants across the first five phases of the IPOS-Dem easy language translation and adaption process

Phase	Number of Participants	Background
I: Conceptual definition	4	Health care professionals
	2	Laypeople
II: Forward translation	2	Easy language specialists
	1	Layperson
III: Backward translation	2	Health care professionals
III: Backward translation comparison	1	Layperson
	4	Health care professionals
IV: Expert review	6	Health care professionals
	2	Easy language specialists
V: Cognitive debriefing	4	Registered nurses
	8	Care assistants
	4	Family members
	2	Laypeople
VI: Proofreading	2	Original IPOS development team members

### Phase V: Cognitive debriefing

The fifth phase assessed how participants understood and conceptualised the IPOS-Dem instructions, questions and scales. After the translation process detailed above, cognitive interviews with two focus groups were conducted in April 2021. Our interviews involved a convenience sample of 10 people from the primary user group, nursing home frontline staff. The focus groups were held face to face and remotely via video call. We outline the participant details in Table 1. As in previous cultural adaptions, we applied the ‘think out loud’ interview method, in which the participants verbalised their thought processes as they answered each survey question [24].

### Phase VI: Proofreading

Detailed documentation of the process and results were submitted to the IPOS-Dem authors for review.

### Setting and participants

Study participants were nurses with clinical and academic backgrounds and certified specialists for easy language recruited from our professional network, as well as people without health care, linguistic or dementia care backgrounds, hereafter called ‘laypeople’. Participating clinical nurses in Phases II and V were recruited from a long-term care facility.

### Study procedures

Participants were asked for written consent with an information leaflet and were provided with a verbal explanation detailing the study and data-handling procedures. Following consent, participants completed the IPOS-Dem pre-final version and were invited to ‘think out loud’. All interviews were recorded using a digital audio recorder or the recording feature of the video conferencing software. During interviews, we asked the participants repeatedly how they understood the individual instructional paragraphs and questions and asked them to verbalise their thoughts and understandings of the IPOS-Dem. If ambiguous or unclear passages were highlighted, we asked participants to suggest a rewording. The recordings of the Swiss-German interviews were transcribed verbatim by the last author.

### Data analyses

The last author analysed the resulting transcripts per item and coded them using ATLAS.ti version 9.1 for MacOS. Then, the first and last authors compared the conclusions of the different groups. Finally, essential parts and remarks were cleaned and collated in tables for each stage.

### Ethical considerations

The study procedures and compliance with applicable research regulations [25, 26] were confirmed by the Zurich cantonal ethics board (BASEC-ID: BASEC2019-01847).

## Results

Besides the findings presented per phase in the paragraphs below, each phase produced and asked for different iterations of the IPOS-Dem. Table 2 illustrates inputs and outputs of each phase we used and referred to below.

**Table 2:** Input and output of intermediary documents for each phase

Phase	Description	Input(s)	Output(s)
I	Conceptual Definition	German IPOS-Dem	
II	Forward translation	German IPOS-Dem	FT1/FT2, FT3
III	Backward Translation	FT3	IPOS-Dem BT1/BT2
IV	Expert review	German IPOS-Dem, English IPOS-Dem, FT3, BT1, BT2	Draft easy language IPOS-Dem
V	Cognitive Debriefing	Draft easy language IPOS-Dem	Pre-final language IPOS-Dem
VI	Proofreading	Pre-final easy language IPOS-Dem	Final easy language IPOS-Dem

### Phase I: Conceptual definition

A few nursing homes applied the Edmonton Symptom Assessment Scale, an instrument validated for symptom assessment and screening in palliative care cancer patients, to people with dementia [27]. However, none of the outcome measurement processes or instruments identified in use were intentionally designed with the target population of people with dementia in mind. Instruments built into the Resident Assessment Instrument for Nursing Homes (RAI-NH) and the Resident Classification and Billing System for Care Services provided (BESA) are more widely used. Nevertheless, the processes attached to these are sub-optimal for the rigorous surveillance of changing and complex symptoms and concerns required in people with dementia. RAI-NH and BESA are only assessed every six months and only by registered nurses qualified to do so [28]. The specialist discussion concluded that an adapted, easy-to-use, and brief multi-dimensional outcome scale for clinical practice is not available.

Discussions with nursing home staff, specialists, laypeople and family members using the German IPOS-Dem concluded that IPOS-Dem concepts are appropriate and well recognised for Swiss nursing homes and dementia care in general. Nevertheless, it was remarked that the German IPOS-Dem was presented in too elaborate a way for the majority of frontline staff to understand [29].

### Phase II: Forward translation of the IPOS-Dem to ‘Leichte Sprache’

In the following paragraphs, we present the changes made with English translations in parentheses.

The forward translations FT1 and FT2 were similar. The significant differences decided on for FT3 were the introductory texts and the first three questions. Long words and words not in everyday use were omitted in favour of shorter and more common synonyms. ‘The person affected’ was changed to ‘person with dementia’ following the Dementia Engagement and Empowerment Project (DEEP) guidance [30].

Sentence structures were adapted according to the easy language rules [31]. The introductory text was expanded to make the questionnaire self-explanatory. It introduced the recall period chosen and led to the general purpose of the questionnaire.

For consistency with the lead-in, a change to the scale was suggested. We translated ‘mäßig’ (‘moderately’) to ‘mittel’ (‘medium’), ‘stark’ (‘substantial’) to ‘Schlimm’ (‘severe’), ‘sehr stark’ (‘very substantial’) to ‘Sehr schlimm’ (‘very severe’) and ‘Nicht beurteilbar’ (‘not assessable’) to ‘Weiss nicht’ (‘don’t know’).

In the symptom list and later questions, ‘z.B.’ (‘e.g.’) was always omitted. For dyspnoea, we added an explanatory sentence in parenthesis. Compound words like ‘Mundtrockenheit’ (‘dry mouth’) were split into their stems, in this case ‘Trockener Mund’. The three translators independently agreed to omit the example ‘z.B. bewusstlos’ (‘e.g., unconscious’) entirely, instead presenting the ‘Weiss nicht’ (‘don’t know’) option for the second set of questions. The question asking for practical problems was revised to enable the use of the same scale used with the preceding questions. It omitted the very uncommonly used term ‘angegangen’ (‘approached’) from all four descriptors.

### Phase III: Backward translation to German

For Phase III, we reported the agreement during the review. In FT3, 37 changes to the original IPOS-Dem were suggested. After reviewing BT1 and BT2 and the original IPOS-Dem, our group achieved good agreement (four out of five members agreed to the change) or better than good agreement for 30 changes. None of the changes were definitively rejected. We discussed items with significant discrepancies and minor changes between BT1 and BT2 and the original IPOS-Dem in the expert group meeting.

### Phase IV: Expert group review

Our group reached at least a fair agreement (five out of eight agreed) or better for all changes in FT3 regarding concepts, semantics, experiential and content equivalence. Controversial items for which our group reached a fair agreement are provided in the Additional file 1. Based on the discussion, a pre-final version was compiled for cognitive interviews.

### Phase V: Cognitive debriefing with staff and specialists

Both focus groups interpreted the questions very well. There were no specific questions or recommendations for rewriting in this stage. The comments regarding comprehension are presented in Table 3. Both groups independently agreed again that the German version of the IPOS-Dem was unclear for the Swiss-German context. They proposed the easy language version to be most acceptable for nurses, frontline staff and family members. Completion time was usually less than 10 min. The group concluded that caring quality, quality of care and needs could be assessed well using the easy language version.

**Table 3:** Comments on modified pre-final IPOS-Dem sections from cognitive interviews

Pre-Final IPOS-Dem	Findings
You are caring for a person with dementia We would like you to tell us: How was the person with dementia doing during the last 7 days? What worried the person with dementia’s family or friends? Which problems did you or someone else encounter during nursing care and social care? Important: All questions always concern the last 7 days Please write clearly	ProInfirmis recommendations are well written and clear: ‘7 days’ instead of the words ‘last week’ is the better alternative
Date today (day/month/year)	‘Date today’ is clearer than just ‘date’
No appetite (don’t want to eat)	Clear; a lot of people with dementia have a poor appetite. The English term is not accurate in the Swiss context
Problems with teeth	The change in the pre-final-version is clear
Is tired or sleepy during the day	Sleepiness is more apparent than drowsiness; explanatory example is not needed
Impaired mobility (trouble walking, cannot get up, falls)	Impaired mobility is apparent, especially in the context of kinesthetics
Difficulty communicating (speaking or any kind of body language)	Addition in the brackets is straightforward and also necessary
Can’t sleep (during the night)	Addition in the brackets is precise and also necessary
Agitation	Discussion about the explanation in the brackets. However, it is not necessary for the Swiss context because of the serial trial intervention assessment (STI)
Has she/he been feeling anxious or agitated?	Changes in the German version are apparent due to the usage of the STI
Do you think she/he felt sad or unhappy?	Sad and unhappy are both necessary
Has she/he been able to talk to others or get in touch in some way (team, family, residents)?	Addition with ‘positively’ is not necessary
If there was a problem, were you able to do something to resolve it? (help with hearing aids, organise foot care or smooth food)	Practical problems are to be considered in the context of everyday life

### Phase VI: Proofreading

The final Swiss-German easy language IPOS-Dem version was endorsed and published by the original developers after proofreading the process report. The Swiss-German IPOS-Dem easy language version can be downloaded and used for free at https://pos-pal.org/maix/ipos-dem.php. The development team for the German IPOS-Dem and English IPOS-Dem wait for psychometric evaluations with the Swiss-German easy language IPOS-Dem version before considering easy language adaptions for their respective versions.

## Discussion

We demonstrated the content and face validity of a Swiss-German easy language version of the IPOS-Dem. In cognitive interviews with specialists and frontline staff, an easy language version was discussed. Frontline staff found the pre-final IPOS-Dem in easy language to be brief and relevant. However, even after the pos-pal core group at the Cicely Saunders Institute of Palliative Care, Policy & Rehabilitation, Florence Nightingale Faculty of Nursing, Midwifery & Palliative Care King’s College London approved the final version of the easy language IPOS-Dem, a psychometric validation of the scale is still warranted.

We encountered challenges translating the IPOS-Dem similar to those described by Sterie and Bernard [32]. Regarding gender-inclusive and non-discriminating language, we improved the questionnaire by continually referring to ‘Mensch mit Demenz’ (‘person with dementia’) throughout the text. Frontline staff in nursing homes refer to people with dementia in many ways: ‘resident’, ‘guest’, ‘client’. In dialect day-to-day communication, clinicians and laypeople also tend to use ‘the demented [person]’, which is considered ableist [33]. Furthermore, all terms like this, when translated into German, always lead to the necessity of gender-inclusive language, resulting in similarly complex sentence constructions, like ‘Bewohner:innen’ or even ‘Bewohnerinnen und Bewohner’ for ‘Residents’. A uniform and handy reference derived from ‘resident’ also seemed contradictory to the original intersectoral approach with IPOS-Dem. Therefore, in the expert rounds, we agreed to use the wording suggested by DEEP [30].

There were other options, most prominently ‘Betroffene Person’ (‘person affected’). We acknowledged the benefit of inclusion, possibly allowing assessment also for older people without a formal diagnosis. However, the German translation resulted in ‘Betroffene Person’, which should be avoided for two reasons when applying the easy language ruleset [31]. First, short words in everyday use are encouraged (we conferred with ProInfirmis that mentioning dementia is acceptable without an explanation since users will know the term because of the setting). Second, ‘affected’ in German implies a negative impact on the person, whereas easy language promotes positive writing.

Popular opinion towards easy language appears ambiguous and was described by Maaß as ‘having an acceptability issue’ [18]. Easy language was developed for inclusion, mainly for people with learning disabilities. Some critics have argued [18] that the open and distinctive presentation of easy language texts may stigmatise the primary target group and further reduce the acceptance of easy language texts. Providing a patient-centred outcome measure in easy language is an example of exposing easy language application to individuals other than people with learning disabilities. Since scales always have a distinctive look, easy language’s possibly disruptive looks and features are somewhat concealed from users.

Easy language enabled us to circumvent the syntactic inconsistencies in items beginning with ‘Do you think…’ right from the beginning. Rephrasing the item regarding practical issues was a strong recommendation by our experts and translators. It now uses the same frequency scale as previous items. In easy language, we do not use the involved and spacious scale ‘problems addressed/no problems’. Because practical problems are to be considered in the context of everyday life, we rephrased it to ‘Wenn es Probleme gab, konnten Sie etwas dagegen tun?’ (‘If there was a problem, were you able to do something to resolve it?’). Surprisingly to the research team, the translation and concept of ‘im Frieden mit sich selbst [sein]’ (‘being at peace’) was taken up very well in all phases and never showed discrepancies in the discussion rounds.

## Conclusions and implications

### Clinical practice

Projected developments in the professional nurse workforce point to local and global shortages in registered nurse human resources [34]. These shortages have repercussions for hospitals but also mandate novel models of care in nursing homes. Frontline staff spend many hours of their shifts directly interacting with people with dementia. While a substantial part of the frontline staff in Swiss nursing homes receives little to no formal training in nursing [35], they provide valuable observations and substantially contribute to caring outcomes. To empower them, routine instruments for their use need to be easily accessible and clinically relevant. Using the information conveyed through IPOS-Dem, an interprofessional team may gain insight to manage further the complex needs of people with dementia living in nursing homes [36].

### Research implications

Easy language may have further applications in patient and public involvement. Its unique qualities enable the development of inclusive and accessible patient-centred outcome measures or self-management pamphlets. The attentive use of easy language communication may also foster increased engagement with untrained laypeople or people with migratory backgrounds in clinical and care research.

## Supplementary Information

**Additional file 1.** Level of agreement regarding concepts, content, experience and semantics during phase IV - expert review.

## Data Availability

Data sharing is not applicable to this article as no datasets were generated or analysed during the current study.

## Abbreviations

BESA: Resident classification and billing system for care services provided (Bewohner:innen-Einschätzungs und Abrechnungssystem)
BT1, BT2: Backward translations
DEEP: Dementia Engagement and Empowerment Project
FT1, FT2 and FT3: Forward translations
IPOS-Dem: Integrated Palliative Care Outcome Scale for People with Dementia
RAI-NH: Resident Assessment Instrument for Nursing Homes
STI: Serial trial intervention
