# Supplementary material for: Integrated Palliative Outcome Scale for People with Dementia: easy language adaption and translation
Source: J Patient Rep Outcomes. 2022 Feb 15;6:14. doi: 10.1186/s41687-022-00420-7 (PMC8847462; doi:10.1186/s41687-022-00420-7)
Supplement: Supplementary file 1 — Additional file 1. Level of agreement regarding concepts, content, experience and semantics during phase IV - expert review. [file 41687_2022_420_MOESM1_ESM.docx]

| **Original IPOS-Dem** | **Group Discussion** | **Agreement** | | | |  |
| --- | --- | --- | --- | --- | --- | --- |
|  |  | **C** | **S** | **E** | **CE** | |
| Please write clearly | The new introduction was a strong recommendation of the Swiss plain language service. | Good | Complete | Complete | Complete | |
| Please write clearly  Person’s name  Person’s number  Date (dd/mm/yyyy) | “Person with dementia” was considered most appropriate. | Good | Complete | Complete | Complete | |
| Q1:  What have been the person’s main problems over the past week? | In the Swiss-German version of IPOS-Dem, “person/people with dementia” instead of “person affected” was used. Therefore, the terms “person/people with dementia” are replaced in the S-GER version.  “Over the past week” is replaced in the S-GER version with “the last 7 days” in a novel introduction. | Good | Fair | Complete | Complete | |
| Not existent in the original version. | Two free-text questions have been added in the German version and reused for the S-GER version. | Complete | Good | Complete | Complete | |
| Q2. Please select one box that best describes how the person has been affected by each of the following symptoms over the past week. | A sentence was added to explain how to rate how much the person with dementia has been affected by the symptom and not how strong the symptom was. Therefore, the added sentence was also left in the Swiss-GER version. | Good | Complete | Complete | Fair | |
| Answer possibilities: not at all, slightly, moderately, severely, overwhelmingly, cannot assess. | Used common words to enhance readability. | Good | Complete | Complete | Fair | |
| Shortness of Breath | Example was needed. | Complete | Complete | Complete | Complete | |
| Nausea (feeling sick/vomiting) | Reduced wordiness to enhance readability. | Complete | Complete | Complete | Complete | |
| Poor appetite | Adding “Don’t want to eat” was considered a better fit. | Complete | Fair | Complete | Complete | |
| Dental problems or problems with dentures | The wording has slightly changed from the German to the Swiss-German version, but not from English to German. | Good | Fair | Complete | Fair | |
| Drowsiness (sleepiness) | The change to a descriptive phrase helps with readability. | Good | Complete | Complete | Good | |
| Poor mobility (trouble walking, cannot leave the bed, falls) | The selection of common words improved readability. | Good | Good | Complete | Fair | |
| Skin breakdown (redness, skin tearing, pressure damage) | We considered “biting” as an often-used slang word for itching, but it was deemed confusing. | Good | Fair | Complete | Fair | |
| Difficulty communicating | An easy language explanation in brackets is consistent with other items and helps with readability. | Complete | Complete | Complete | Complete | |
| Sleeping problems | The change to the descriptive phrase helps with readability. | Good | Complete | Complete | Fair | |
| Hallucinations (seeing or hearing things not present) and/or delusions (fixed false beliefs) | The selection of common words improved readability. | Complete | Complete | Complete | Complete | |
| Agitation (restless, irritable, aggressive) | Explanation of the term was left out in the GER version and also in the S-GER version. | Complete | Complete | Complete | Complete | |
| Wandering (as a result of distress or putting person at risk) | In Swiss-German, the term “distress” is not used in the nursing home/care home setting. | Complete | Complete | Complete | Complete | |
| Answer possibilities: not at all, occasionally, sometimes, most of the time, always, cannot assess. | The selection of common words improved readability. | Good | Complete | Complete | Fair | |
| Q3:  Has s/he been feeling anxious or worried? | Worried can be left off because, in the S-GER version, the term “agitated” is more precise to the English meaning. | Complete | Complete | Complete | Complete | |
| Q4:  Have any of his/her family been anxious or worried about the person? | “Friends” needed to be added because of the family nursing concept. Family and friends are considered one group accordingly.  The word “worried” was left off because it has the same meaning as being anxious in Swiss-German. | Complete | Complete | Complete | Complete | |
| Q5:  Do you think s/he felt depressed? | The selection of common words improved readability.  Participants in the forward translation for the Swiss-German IPOS-Dem felt that “or” needs to be included because “sad” and “depressed” have two different German meanings within the same overarching topic: feeling sad. | Complete | Complete | Complete | Complete | |
| Q5b:  Lost interest in things s/he would normally enjoy? | The change to a descriptive phrase helps with readability. | Fair | Complete | Complete | Good | |
| Q6:  Do you think s/he felt at peace? | The change to a descriptive phrase helps with readability and consistency. | Complete | Complete | Complete | Complete | |
| Q7: Has s/he been able to interact positively with others (e.g., staff, family, residents)? | The change to a descriptive phrase helps with readability and consistency. | Good | Complete | Fair | Complete | |
| Q7b:  Can s/he enjoy activities appropriate for his/her level of interest and abilities? | This question has been left off in the GER version and also in the S-GER version. | N/A | N/A | N/A | N/A | |
| Q8:  Has his/her family had as much information as desired? | This question has been left off in the GER version and also in the S-GER version. | N/A | N/A | N/A | N/A | |
| Not existent in the original version.  Was s/he irritated or aggressive? | The change to a descriptive phrase helps with readability and consistency. | Complete | Complete | Complete | Good | |
| Q9:  Have practical problems been addressed? (e.g., hearing aids, foot care, glasses, diet) | This question caused the most translation problems (forward and backward) because the meaning is different in S-GER. | Complete | Fair | Complete | Complete | |
| Not existent in the original version.  Q9b:  If not: What was the reason? | It helps to consider Q9 in day-to-day practice. | Complete | Fair | Complete | Complete | |
| What was the person’s last weight and the date s/he was last weighed?  Weight: … kg  Date: …/…/… | This question has been left off in the GER version and also in the S-GER version. It is usually captured in minimum datasets. | N/A | N/A | N/A | N/A | |

**Table S1: Level of agreement regarding concepts, content, experience and semantics during phase four - expert review.**

Complete agreement: all eight expert group members agreed; Good agreement: six to seven members agreed; Fair agreement: five members agreed. Abbreviations: C: Conceptual; CE: Content equivalence; E: Experiential; GER: German; N/A: Not available; S: Semantic; S-GER: Swiss-German.
